# Using Natural Biomacromolecules for Adsorptive and Enzymatic Removal of Aniline Blue from Water

**DOI:** 10.3390/molecules23071606

**Published:** 2018-07-02

**Authors:** Xiaojuan You, Enzhong Li, Jiayang Liu, Songhua Li

**Affiliations:** 1 Fermentation Technology Division, Huanghuai University, Zhumadian 463000, China; youxiaojuan1988@163.com (X.Y.); enzhongli@163.com (E.L.); 2 Henan Dongfangrun Environmental Protection Technology Co. Ltd., Zhumadian 463000, China; dfrhbkj@163.com

**Keywords:** adsorption, adsorbent, laccase, aniline blue, dye removal

## Abstract

The present study investigated the adsorptive and enzymatic removal of aniline blue dye (AB) from aqueous solution using waxy riceprocessing waste (RW), peanut shell (PS), microbial waste of *Aspergillus niger* (MW) as low cost adsorbents, and laccase (Lac) as a biocatalyst. Commercial activated carbon (AC) was also employed to compare the adsorption performance with the three adsorbents. Dye removal was examined under various parameters in batch experiments. It was found that dye removal by RW and Lac was 89–94% noticeably better than that by MW and PS (20–70%). In any cases, AC produced the highest dye removal among the tested materials. The kinetics, isotherms, and thermodynamics were then analyzed to elucidate the adsorption process by the four adsorbents. The pseudo-second order kinetic was superior to the pseudo first order kinetic model in describing adsorption for all adsorbents. The Langmuir model fitted the adsorption process very well, indicating monolayer coverage of dyes on a solid surface. A thermodynamic analysis of enthalpy (Δ*H*°), entropy (Δ*S*°), and Gibbs free energy (Δ*G*°) classified the adsorption as a nonspontaneous and endothermic process. The results reveal diverse natural materials (e.g., processing waste RW) as novel substitutes for traditional activated carbon, as well as laccase as a green catalyst for the treatment of dye wastewater.

## 1. Introduction

The unprecedented growth of human society and industrialization has caused a striking negative impact on the earth’s ecosystem [1]. The main water pollutants typically encompass inorganic metal ions and organic compounds with a variety of tough dyes [2]. To date, the treatment of dye wastewater relies on some developed schemes including a biological, a physical, and a chemical method, and/or a combination of these—each of which has its advantages and disadvantages [3,4,5]. Adsorption and enzymatic treatment are thought to be both effective and eco-friendly in terms of removing dyes from wastewater, due to their low process cost and short process time [3]. A series of substances have been attempted for adsorption purposes, e.g., agricultural waste peels, activated carbon, synthetic material, microbial bacteria, and natural-minerals, with varied adsorption performance and behavior obtained [6,7,8]. Compared with cultivation-based biological methods which possibly increase the COD (chemical oxygen demand) of the wastewater due to nutrient supplementation, enzyme-mediated degradation of dyes seems to be more efficient and clean [9].

For adsorption technology, activated carbon has been developed into a mature and extensively used material in wastewater treatment [10,11]. Despite the existing achievements, seeking novel, cheap alternatives is still of great importance because real industrial wastewaters always come in extreme diversity, not only in discharge amount, but also in its composition [12]. In this study, we collected three different wastes from either a local farm or company, and one home-made enzyme of laccase in our lab. We then evaluated their potentials in removing dye from water with aniline blue as the model dye. Rice waste (RW) was collected from sediment in a wastewater tank that was affiliated to a waxy rice processing and production base (Xinyang, China). Peanut shell (PS) was provided by a peanut plantation and production base (Zhumadian, China). Microbial waste (MW) of *Aspergillus niger* after fermentation for amylase production was provided by Henan Yangshao Bioengineering Co., Ltd. (Sanmenxia, China). These wastes represent the typical wastes across the Henan Province that currently require certain capital and labor input for their treatment. Meanwhile, these wastes are composed of some of biomacromolecules like cellulose, hemicellulose, lignin, pectin, glucan, protein, and starch, which have a series of active functional groups and thus, might serve as effective adsorbents [13]. In response to the call for sustainable development, these wastes should be properly treated and/or recycled as novel value-added materials with other potential usages. Copper-containing laccase as a type of polyphenol oxidase has been extensively reported on and shown to have numerous applications in a number of industrial and agricultural fields, including dye effluent treatment [14,15]. After treatment with laccase, the chromaticity and toxicity of industrial wastewater are greatly decreased [14].

Aniline blue is a type of triarylmethane dye with wide applications such as in dying cells for medicinal purposes, and only few studies have been focused on removing it from water [16]. In this study, RW, MW, PS, and Lac were used as natural biomacromolecules to remove aniline blue from water via an adsorptive and enzymatic approach. To make comparisons, commercial activated carbon was also employed. First, dye removal efficiency was examined with the four adsorbents and one enzyme under various conditions. Then, the adsorption behaviors were modeled with equilibrium, thermodynamic, and kinetic equations.

## 2. Materials and Methods

### 2.1. RW, PS, MW, AC, Lac, and Aniline Blue

The sediment/slurry was taken from the bottom of a wastewater tank just downstream, which followed the discharge pipe in a waxy rice processing plant (Xinyang, China). The collected rice waste (RW) was oven dried at 60 °C and was then ground into powders to pass through a 100-mesh screen. RW consists of rice bran, rice husk, and rice residual. PS was collected from a farmland in Zhumadian City, Henan Province, China and was washed with tap water to eliminate impurities on the surface. This was followed by being dried in an oven at 60 °C. The completely dried material was cut into small pieces using scissors and was ground and sieved using a 100-mesh screen. PS contains cellulose (40.5%), hemicellulose (14.7%), and lignin (26.4%) [17]. MW was collected from Henan Yangshao Bioengineering Co. Ltd., Sanmenxia City, Henan Province, China. The fresh residue from after the fermentation and amylase separation was soaked in distilled water for 4 h to remove the possible impurities and was then dried at 60 °C. MW is composed of *Aspergillus niger* mycelial pellet, perlite, and diatomite. All of the above dried samples were stored in an airtight vessel at room temperature and were directly used for adsorption experiments without further treatment. Commercial activated carbon (AC) was purchased from a local market.

*Pycnoporus* sp. SYBC-L3 (GenBank access number JX861099) is a well-defined ligninolytic fungus for hyper laccase production and was used herein to produce laccase through a previously identified method [18]. The fungus was maintained on potato dextrose agar (PDA) plates at 4 °C and was periodically transferred for activation every two weeks. Lac by fungus L3 is a copper-containing oxidoreductase with a molecule weight of 58 kDa [15].

**Figure 1:**
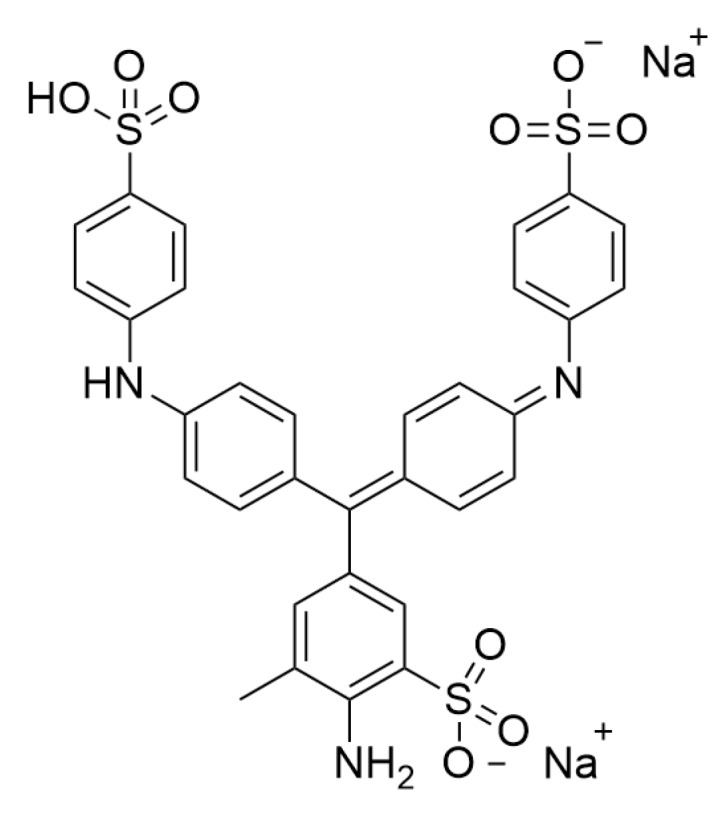
The chemical structure of aniline blue.

Aniline blue (C_37_H_32_N_5_O_9_S_3_, 786.9 g/mol, Figure 1), also known as Potassium nonafluoro-1-butanesulfonate, is an acid mixed dye and was purchased from Sinopharm Chemical Reagent Co., Ltd. (Shanghai, China). This dye is widely used in the textile industry for dyeing wool, silk, and wool blends as well as in dying nerve tissue, cell, and junction tissues during biological studies. In these applications, a certain amount of aniline blue wastewater is produced. Aniline blue solution (50 mg/L) was scanned via a U-300 UV-Vis Spectrophotometer (Shimadzu Corporation, Kyoto, Japan) to obtain a wavelength for maximal absorption. Based on the wavelength, the standard curve of AB was made for the following dye concentration determination.

### 2.2. Adsorption Experiments

Dye adsorption was conducted in a 250 mL flask containing 50 mL dye solution as a function of dye concentration, adsorbent dose, contact time, temperature, and NaCl concentration under the condition of a rotation shaking speed of 100 rpm. After adsorption, the solution in each flask was centrifuged at 4000 rpm for 10 min to obtain the supernatant that was used to determine the dye concentration. The dye removal rate (1) and the dye adsorption amount at equilibrium (*q_e_*) (2) were calculated as follows:

(1) $$R=\frac{c_{0}-c_{e}}{c_{0}}\times 100\%,$$

(2) $$q_{e}=\frac{{(c}_{0}-c_{e})\times V}{W}$$

where *c*_0_ represents the initial dye concentration (mg/L), *c_e_* represents the equilibrium dye concentration (mg/L), V represents the volume of the dye solution (L), W represents the weight of the adsorbent (g), and *q_e_* represents the equilibrium adsorption amount (mg/g). All sorption experiments were performed in triplicate with relative standard deviations of <5%, and the averages for the triplicate data are reported in the results section.

### 2.3. The Effect of Influencing Factors on Dye Removal

Five variables, i.e., adsorbent/enzyme dosage, contact time, dye concentration, temperature, and NaCl were studied concerning their effect on dye removal. One factor at one time method was adopted, while the other factors remained constant. First, adsorbent/enzyme was examined by varying the dosage/activity, beginning from 0.1 g/L or U/mL with dye 50 mg/L, contact time 4 h, and a temperature of 25 °C, from which a relative best dosage for each was identified: RW 4 g/L, MW 1 g/L, PS 2 g/L, AC 1 g/L, and Lac 1 U/mL. This best dosage was then used for the following investigation. Dye adsorption was conducted for various contact periods within 12 h, with dye concentration 50 mg/L, temperature 25 °C, and a dosage from the above. Dye adsorption was conducted by varying the initial dye concentration in the range of 20–400 mg/L at a contact time of 4 h, a temperature of 25 °C, and a dosage from the above. Dye adsorption was conducted by varying the temperature from 10 to 60 °C at a contact time of 4 h, an initial dye concentration of 50 mg/L, and an adsorbent dosage from the above. Dye removal was also studied in the presence of NaCl, ranging from 0.1 to 1 mol/L.

### 2.4. Adsorption Kinetic Studies

After adsorption under various contact times (see above), the dye removal rate and equilibrium adsorption capacity (*q_e_*) were calculated as stated above. The data was then fitted with Lagergren’s pseudo first order model (3) and Ho’s pseudo second order model (4).

(3) $$\mathrm{lg}\left(q_{e}-q_{t}\right)=-\frac{K_{1}t}{2.303}+\mathrm{lg}q_{e}$$

(4) $$\frac{t}{q_{t}}=\frac{t}{q_{e}}+\frac{1}{K_{2}q_{e}{}^{2}}$$

where *q_e_* represents the equilibrium dye adsorption amount (mg/g), *q_t_* represents the adsorption amount (mg/g) at contact time *t* (h or min), *K*_1_ represents the equilibrium rate constant of the first order sorption (min^−1^), and *K*_2_ represents the equilibrium rate constant of the second order sorption (g/mg·min).

To identify the adsorption mechanism, the adsorption data was also fitted with both the film diffusion model (5) and the intraparticle diffusion model (6).

(5) $$\ln\left(1-\frac{q_{t}}{q_{e}}\right)=-K_{3}t$$

(6) $$q_{t}{=K}_{4}t^{1/2}+C$$

where *q_e_* represents the equilibrium dye adsorption amount (mg/g), *q_t_* represents the adsorption amount (mg/g) at contact time *t* (h or min), *K*_3_ represents the equilibrium rate constant (min^−1^), *K*_4_ represents the intraparticle rate constant (mg/g·min^1/2^), and C represents the film diffusion extent (mg/g).

### 2.5. Adsorption Thermodynamics

After adsorption under different temperatures (see above), the dye removal rate and equilibrium adsorption capacity (*q_e_*) were calculated as stated above. The change in free energy (Δ*G*°) was evaluated using the following equation to study the thermodynamic nature:

(7) $$∆G^{{}^{\circ}}=-RT\ln\left(\frac{q_{e}}{c_{e}}\right),$$

where *R* represents the gas constant (8.3143 J·mol^−1^·K^−1^), T represents the absolute temperature (Kelvin), *q*_e_ represents the equilibrium adsorption amount (mg/g), and *c_e_* represents the equilibrium dye concentration (mg/L). From a plot of Δ*G* vs. *T*, the value of enthalpy Δ*H* and entropy Δ*S*° can be calculated as follows (8).

(8) $$∆G^{{}^{\circ}}=∆H^{{}^{\circ}}-T∆S^{{}^{\circ}}$$

### 2.6. Adsorption Isotherms

After adsorption under the different initial dye concentrations, the dye removal rate and equilibrium adsorption capacity (*q_e_*) were calculated as stated above. The Langmuir (9) and Freundlich equations (10) were employed to explicate the adsorption isotherms of dye AB on the adsorbents. The linearized forms of the Langmuir and Freundlich equations are as follows:

(9) $$\frac{C_{e}}{q_{e}}=\frac{1}{K_{a}q_{m}}+\frac{C_{e}}{q_{m}}$$

(10) $$\mathrm{lg}q_{e}=\frac{1}{n}\mathrm{lg}c_{e}+\mathrm{lg}K_{F}$$

where *K_α_* (L/mg) represents the Langmuir adsorption constant and *q_m_* (mg/g) represents the maximum dye amount of adsorption corresponding to complete monolayer coverage on the surface, *q_e_* (mg/g) represents the amount of dye adsorbed by sorbent at equilibrium, and *C_e_* (mg/L) represents the equilibrium concentration of dye solution. *K_F_* represents an indicator of adsorption capacity (mg/g) and 1/*n* represents the adsorption intensity. The separation factor *R_L_* was calculated using the following Equation (11) in which *C*_0_ represents the initial dye concentration (mg/L):

*R_L_*= 1/(1 + *KαC*_0_) (11)

## 3. Results and Discussions

### 3.1. Spectrophotometric Calibration

**Figure 2:**
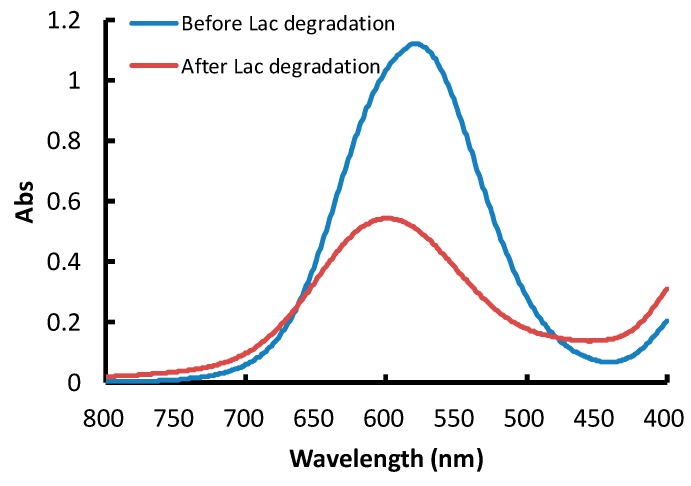
Wavelength scanning of aniline blue solution (50 mg/L) before and after laccase (0.2 U/mL) degradation for 10 min.

The maximum absorbance of AB solution (50 mg/L) was obtained at 581 nm (Figure 2), which was similar to a previous report [9,16]. The standard curve was determined as: y = 0.023x + 0.006 (*R*^2^ = 0.998). With Lac application, the absorption peak was slightly transferred to around 600 nm after 10 min of degradation, and no new absorption peak was found in the visible range. This indicated that Lac could efficiently degrade AB dye into colorless products in the solution. 

### 3.2. The Effect of Influencing Parameters on Dye Removal

**Figure 3:**
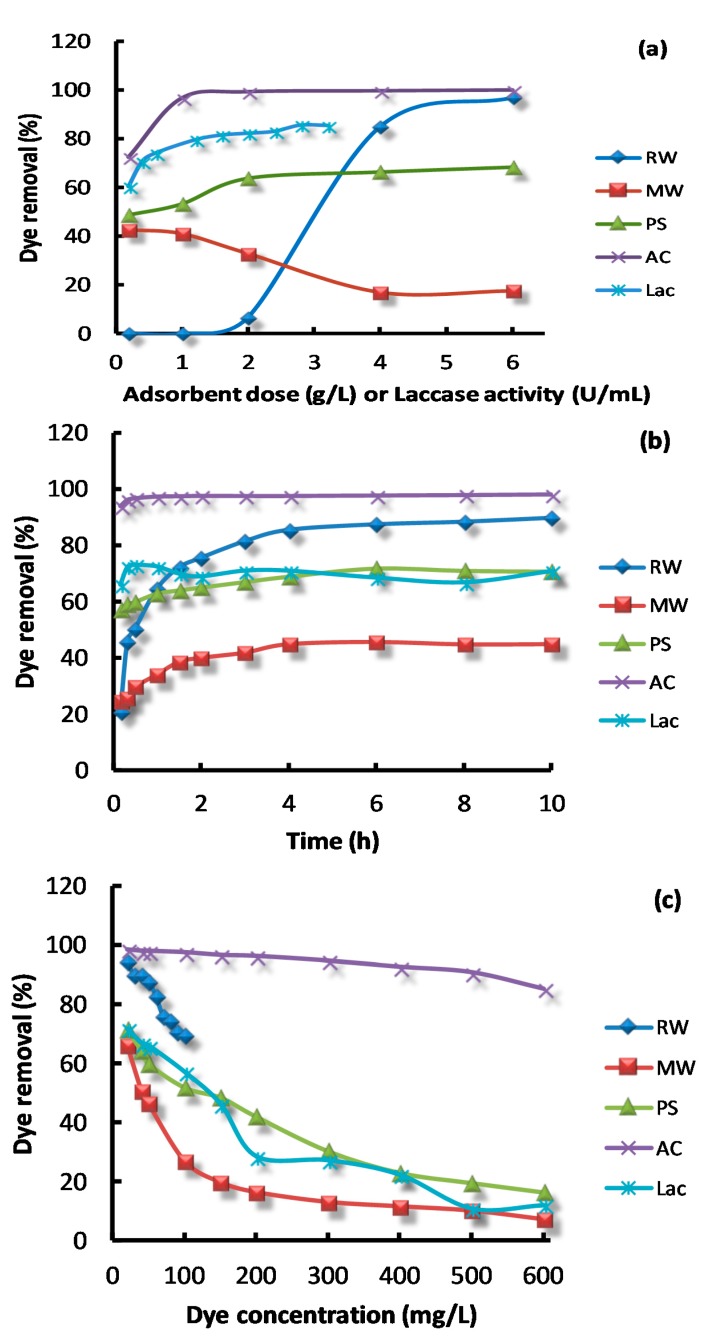

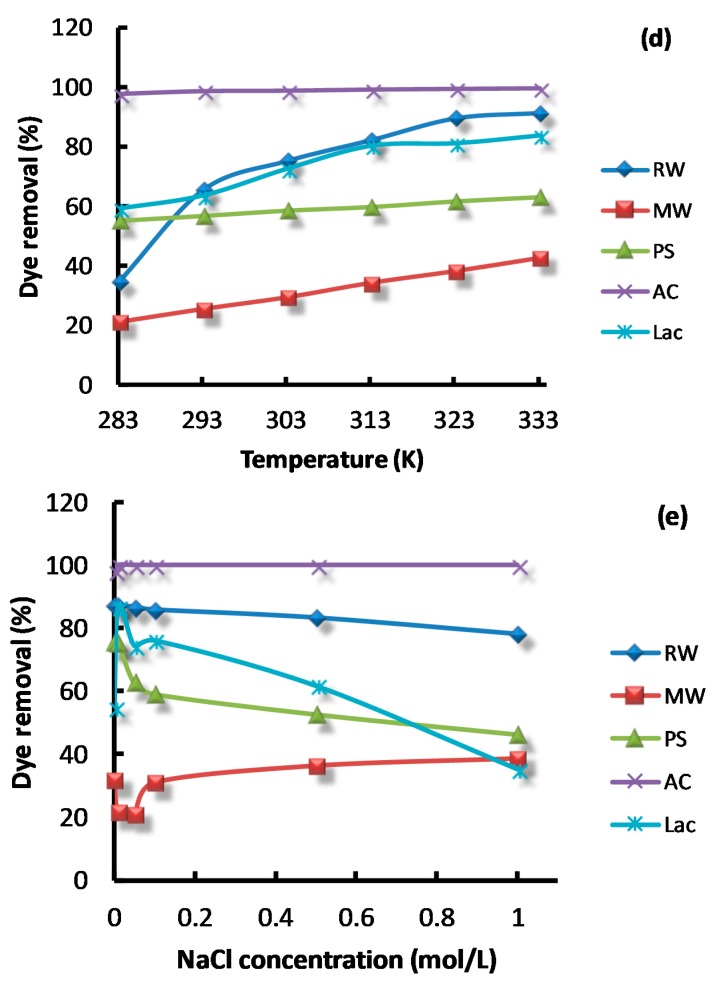
Dye removal under various conditions: adsorbent dose or laccase activity (**a**); contact time (**b**); dye concentration (**c**); temperature (**d**); NaCl concentration (**e**).

Adsorbent dose drastically affects the dye removal efficiency from aqueous solution with a given initial dye concentration. Different adsorbent dosages of 0.2, 1, 2, 4 and 6 g/L were applied to study the corresponding effect on adsorption. As shown in Figure 3a, the tested materials differed in their capability in the corresponding dye removal rate. A significant increase in the percentage of dye adsorption was observed with increasing RW, PS, and AC from 0.2 g to 6 g, which could be attributed to the increase in the availability of adsorption sites on adsorbent surface with the increasing dose of the adsorbents. A maximum of 70%, 94% and 96% of AB dye removal were obtained for PS, RW, and AC at a dose of 2 g/L, 4 g/L and 1 g/L, respectively. Further increases in adsorbent dosage did not promote a significant increase in the percentage of dye removal. This observation might be attributed to the overlapping or aggregation of the adsorption sites, which decreased the total surface area of adsorbents and thus, limited the availability of active sites during the adsorption process [19]. It is obvious that AC showed the highest performance among all the adsorbents, while MW was the worst with less than 40% removal at all dosages. It is noteworthy that besides physical dye removal by adsorbents, enzymatic dye removal by Lac displayed satisfactory results as well. For example, a maximum of 89% of AB dye removal was obtained at a 1 U/mL dose of Lac. From above, RW and Lac are promising agents for AB removal if you are not taking AC into consideration.

The effect of contact time was studied in the range of 0.1–10 h, and the result can be seen in Figure 3b. It was observed that dye removal by the five materials was initially very fast and equilibrium was almost achieved within 1–3 h, which was similar to other adsorbents [20]. A much shorter time of only 10 min was needed for AC and Lac to reach the highest dye removal rate. A further increase in contact time beyond 4 h did not enhance the dye removal. A maximum of 78%, 78%, 92%, 41% and 97% of AB dye removal rate within a varied incubation time was obtained for Lac, PS, RW, MW and AC, respectively. Rapid adsorption occurred at the initial stage mainly because of the strong interactions between the dye molecules and the surface active sites of the adsorbent. However, as the surface sites were gradually more and more occupied and free dye molecules in the solution decreased, the adsorption process gradually slowed and stabilized [21]. Similar results were found for the process of laccase degradation at the initial phase, where the enzyme degraded the dyes very fast because it was not saturated with the substrate of the dye molecules.

The dye concentration in the range of 20–600 mg/L was studied to evaluate its effect on dye removal (Figure 3c). By increasing the initial dye concentration, the dye removal rate decreased significantly, which was similar to most studies [22]. The magnitude of the reduction of the dye removal rate was the least for AC, but was the most for the other agents. To be specific, the application of AC still obtained 90% dye removal at 600 mg/L, while less than 20% was removed for the others at the same dye concentration. This might be caused by the limited adsorptive sites on the adsorbents and the degradation center of Lac that could not hold too many dye molecules.

The effect of temperature was studied in the range of 283–333 K for AB adsorption. From Figure 3d, with increasing temperatures, the dye removal rate was gradually increased and then stabilized, except for AC. This indicated an endothermic characteristic of the adsorption process. Usually, increasing the environmental temperature increases molecular motion and the surface energy of the adsorbent, thus improving the dye removal rate. As for the enzymatic reaction, increasing the temperature could exert both an enhancing enzyme catalytic rate and the inactivation of the enzyme’s original activity. Because Lac by *Pycnoporus* sp. SYBC-L3 was proved to be high-temperature tolerant in our previous study [15], it is not surprising that a higher AB removal rate was still reached at 60 °C.

Industrial wastewater normally contains a large number of inorganic salts, which may affect adsorption in a notable manner, and the salinity tolerance of the adsorbents is one of the important factors affecting the dye removal rate. The effect of NaCl concentration was studied for AB removal, with the result shown in Figure 3e. In general, NaCl significantly influenced dye removal by PS, RW, and Lac in a negative manner. The dye removal rate for AC and MW stayed unaffected throughout all of the concentrations of NaCl that were tested. Lac was most heavily affected because over half of its initial dye removal rates were reduced when the NaCl content increased from 0 to 1 mol/L, showing that NaCl might be an inhibitor of Lac. As for the effect on adsorption, the possible mechanism is that increasing Cl^-^ in combination with the existing anionic dye AB might competitively contact ionic active sites on different materials, which was unfortunately uncharacterized and not provided herein. 

The removal of aniline blue (AB) from water has been previously tested with different methods. Through a combined mechanism of “adsorption-flocculation”, AB was removed from water by 56.5% at 30 min using extracellular polymeric substances (EPS) [23]. A maximum AB removal of 98% was obtained under optimized conditions using a response surface methodology (RSM) with laccase from *Trametes trogii* in the presence of mediator 1-hydroxybenzotriazole (HBT) [24]. Also, with a mediator, a spore laccase from *Bacillus vallismortis* fmb-103 degraded AB up to 81.2% for a 24 h reaction [9]. The addition of 58 kg sodium tetraborate-modified Kaolinite clay would have a capability of adsorbing 95% AB from 1 ton of its solution at a dye concentration of 30 mg/L [16]. 

### 3.3. Adsorption Kinetics

**Figure 4:**
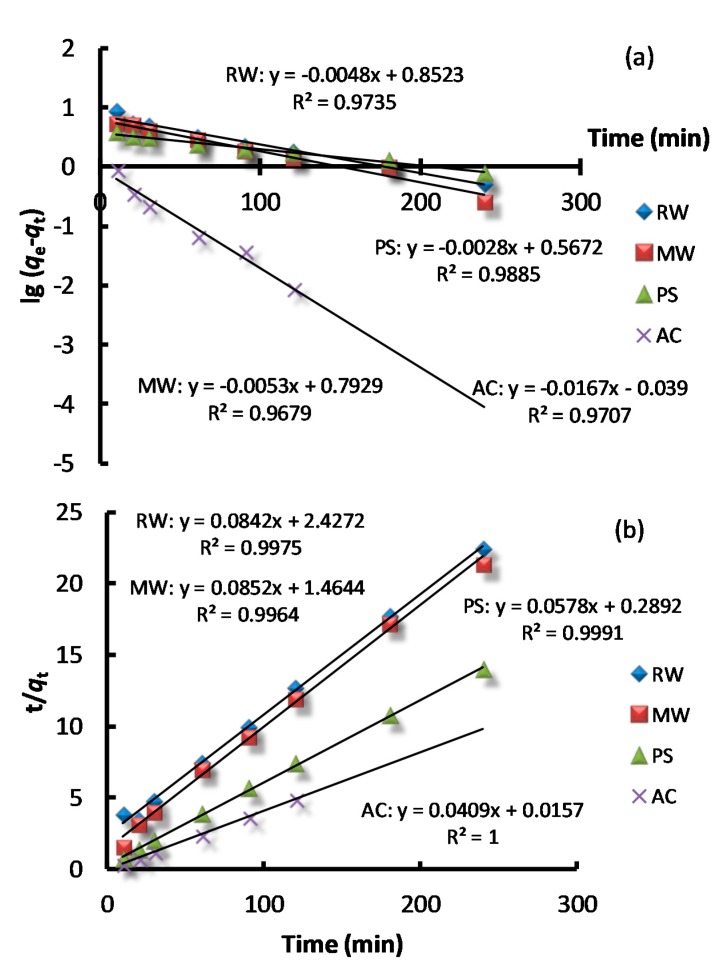

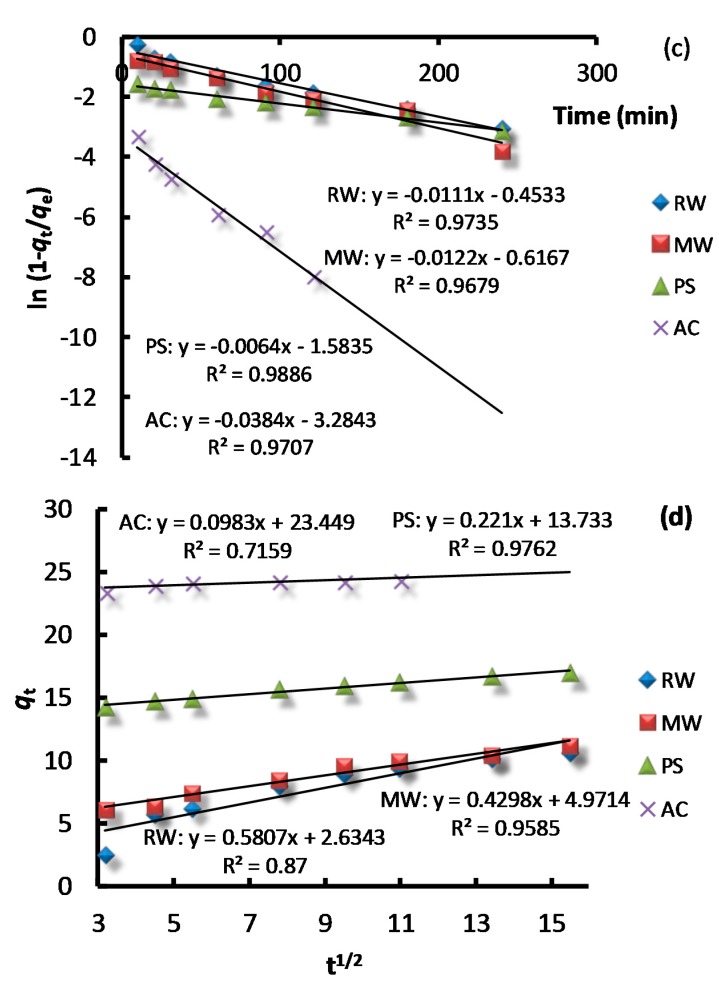
Pseudo first order kinetic (**a**) and pseudo second order kinetic models (**b**); Film diffusion (**c**) and intraparticle diffusion models (**d**).

Two kinetic and diffusion models (pseudo first order, pseudo second order, film diffusion, and intraparticle diffusion model) have been employed for the analysis of kinetic sorption data using the linear regression method. Linear plots for each kinetic model were drawn in Figure 4. Kinetic parameters that were obtained via the linear regression method for the pseudo first order and pseudo second order kinetic models are listed in Table 1.

The kinetic equations in Figure 4a,b show that the pseudo second order kinetic model could better describe the adsorption process (*R*^2^ = 0.99) than the pseudo first order kinetic model (*R*^2^ = 0.97–0.99). The calculated *q_e_*_,cal_ values that were obtained via the pseudo second order kinetic model were found to be closer to the experimental values *q_e_*_,exp_, which indicated the applicability of the pseudo second order kinetic model for the RW, MW, PS, and AC adsorption systems. The pseudo first order kinetics suggests the physical adsorption as the speed limiting step, and pseudo second order kinetics suggests the chemical adsorption as the speed limit step [21]. Therefore, the adsorption of AB dye onto four different adsorbents included both physical adsorption and chemical adsorption, and the chemical reaction was the main rate-controlling step throughout most of the adsorption process.

By comparing the correlation coefficient in two diffusion models, the film diffusion model (*R*^2^ > 0.97) could better describe the adsorption of AB by RW, MW, PS, and AC than the intraparticle diffusion model (Figure 4c,d). It can be deduced that the adsorption process might adopt a very rapid dye adsorption to the external surface and then a slow intraparticle in the interior of the four adsorbents which are all porous materials, despite their different chemical compositions. These results of the kinetic and diffusion models show consistence with the previous findings for the removal of AB using iodo polyurethane foam and *Salvadorapersica* as adsorbents [25,26].

### 3.4. Adsorption Isotherms

**Figure 5:**
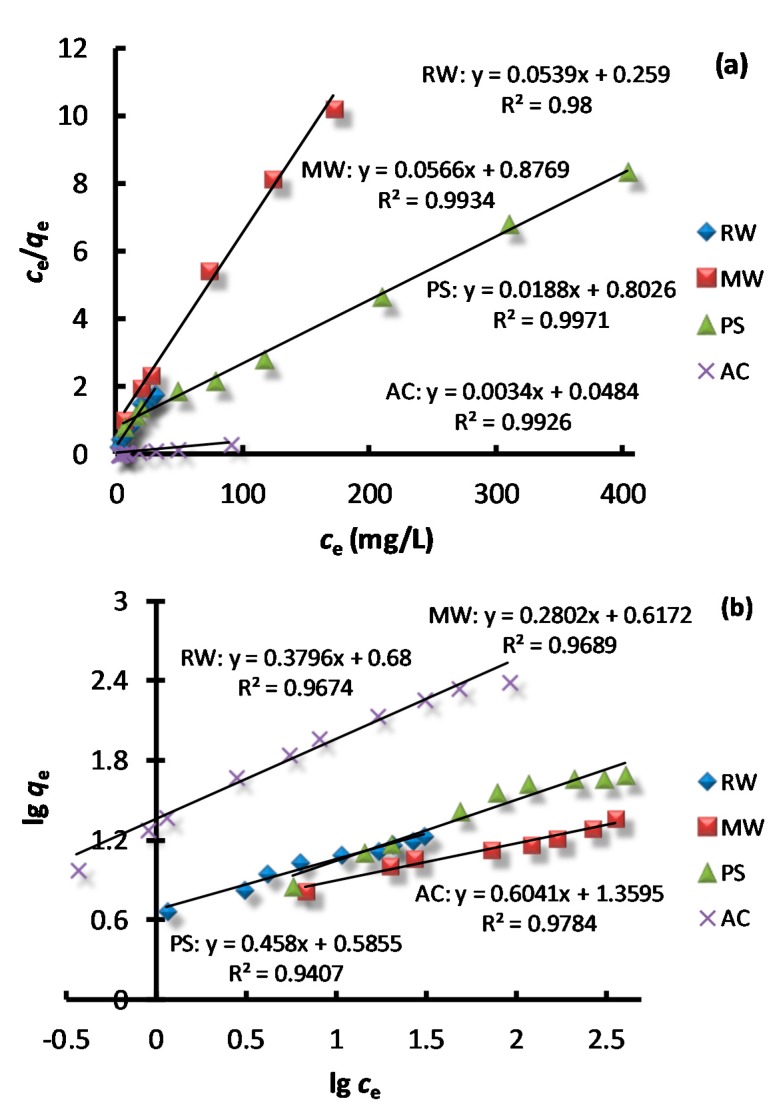
Langmuir (**a**) and Freundlich model (**b**) of adsorption.

Adsorption isotherms represent the specific relationship between the equilibrium concentration of the adsorbent in the bulk and the adsorbed amount at the surface. Plots of Langmuir and Freundlich isotherms are shown in Figure 5, and calculated parameters are listed in Table 2. The Langmuir and Freundlich isotherm models describe adsorption occurring on homogeneous and heterogeneous surfaces, respectively.

In the two adsorption isotherms, the Langmuir model (*R*^2^ > 0.99) could better describe the adsorption process, showing that the adsorption of AB dye from an aqueous solution to the solid surface of four adsorbents was a monolayer coverage, which was identical to the adsorption of *Salvadorapersica* [26] and sodium tetraborate-modified Kaolinite clay adsorbents [16]. As can be seen from Table 2, the largest monolayer adsorption capacity of AB by RW, MW, PS, and AC was 18.55, 17.67, 52.91 and 294.12 mg/g, respectively. It is obvious that AC has a higher adsorption capacity than natural materials. Table 2 also shows that dimensionless separation factor *K_L_* for all of the adsorbents was below 0.5, indicating that adsorption was favorable (0 < *K_L_* < 1). In the Freundlich model, 1/*n* values for the four adsorbents were all less than 1, suggesting that the sorption on the peanut shell was also very feasible [20].

### 3.5. Adsorption thermodynamics

**Figure 6:**
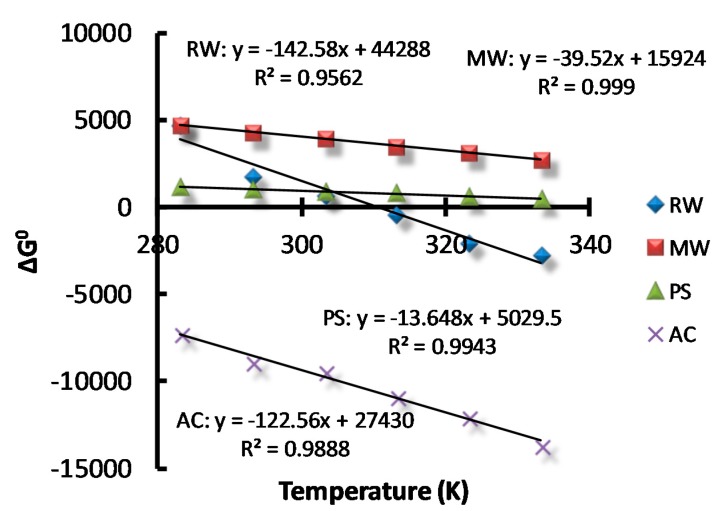
Plot of ΔG° vs. temperature.

ΔG° was calculated and the corresponding values under the different solution temperatures are reported in Figure 6. Negative values of ΔG° indicate that the adsorption of AB dye on AC was feasible and spontaneous in nature [22]. For the adsorption of AB on RW, the temperature profile generated both negative values ΔG° (T > 310 K) and positive values ΔG° (T < 310 K). By contrast, positive values of ΔG° were obtained for the adsorption of AB on MW and PS. ΔG° > 0 indicates that the adsorption process cannot be spontaneous in the standard state. However, in the actual adsorption process, the system was not in the standard state; therefore, the adsorption reaction could still occur.

ΔS° and ΔH° were calculated from the Equation (8) based on the plot of ΔG° vs. temperature in Figure 6, and the numeric values are listed in Table 3. By linear fitting, the *R*^2^ values of the equations were found to be around 0.99 for AC, PS, and MW with the exception of that for RW (*R*^2^ = 0.956). ΔS° reflects the randomness in the internal state of the adsorption system, and larger values of ΔS° responds to the higher degree of randomness [20]. As can be seen in Table 3, ΔS° in the adsorption process was positive, indicating that the adsorption of AB on the surface of the adsorbent was random and not sequential [21]. Moreover, the positive values of ΔS° also illustrated a good affinity between the adsorbent and AB dye. All of the calculated ΔH° values were above zero, which further contended that adsorption was an endothermic reaction. Since most industrial effluents contain a certain amount of heat, and this heat might potentially promote the adsorption reaction [19], the overall treatment efficiency and operation cost could be reduced to some extent.

## 4. Conclusions

The present study clearly demonstrated that some biomacromolecules that are generated from agricultural and industrial waste can be novel green, low-cost adsorbents or biocatalysts for industrial waste water treatment. Among all of the materials tested, AC exhibited the strongest efficiency regarding dye removal (>98%), followed by RW (94%), enzyme Lac (89%), and PS (65%). The Langmuir model could better describe the adsorption process, showing that the surface properties of the adsorbent wereuniform. Dye removal reached its highest within 3 h, and the adsorption process was consistent with the pseudo-second order kinetic model. The increasing temperature could slightly elevate the adsorption efficiency,and the adsorption was an endothermic and spontaneous reaction. The presence of NaCl had an inhibitory effect on the adsorption of AB by adsorbents and the degradation by Lac. Since these wastes represent eco-friendly and inexpensive natural biomacromolecules, they should find broad, practical applications depending on types of wastewater, and could perhaps act as substitutes of AC from the point of view of total cost.

## Figures and Tables

**Table 1:** Parameters for pseudo first-order and pseudo second-order models.

Adsorbent	Pseudo First Order Kinetic Model		Pseudo Second Order Kinetic Model		Experimental
	*K*_1_ (min^−1^)	*q_e_*_(cal)_ (mg/g)	*K*_2_ (g/mg·min)	*q_e_*_(cal)_ (mg/g)	*q_e_*_(exp)_ (mg/g)
RW	0.001	7.12	0.003	11.88	11.08
MW	0.012	6.09	0.016	11.74	11.25
PS	0.007	4.03	0.014	17.21	17.91
AC	0.09	0.91	0.12	24.45	24.52

*q_e_*_(cal)_ is the equilibrium adsorption capacity by calculation; *q_e_*_(exp)_ is the equilibrium adsorption capacity by experiment.

**Table 2:** Parameters for the Langmuir and Freundlich models.

Adsorbent	Langmuir			Freundlich		
	*q_m_* (mg/g)	*K_L_*	*R* ^2^	1*/n*	*K_F_*	*R* ^2^
RW	18.55	0.21	0.98	0.38	4.79	0.967
MW	17.67	0.06	0.993	0.28	4.20	0.969
PS	52.91	0.023	0.997	0.46	3.89	0.941
AC	294.12	0.07	0.993	0.60	22.89	0.978

**Table 3:** Thermodynamic parameters for AB adsorption.

Adsorbent	ΔH° (kJ·mol^−1^)	ΔS° (J·mol^−1^·K^−1^)
RW	44.29	142.58
MW	15.92	39.52
PS	5.03	13.65
AC	27.43	122.56
